# Draft Genome Sequence of a Divergent Anaerobic Member of the *Chloroflexi* Class *Ardenticatenia* from a Sulfidic Hot Spring

**DOI:** 10.1128/genomeA.00571-18

**Published:** 2018-06-21

**Authors:** L. M. Ward, S. E. McGlynn, W. W. Fischer

**Affiliations:** a Department of Earth and Planetary Sciences, Harvard University, Cambridge, Massachusetts, USA; b Earth-Life Science Institute, Tokyo Institute of Technology, Meguro, Tokyo, Japan; c Division of Geological and Planetary Sciences, California Institute of Technology, Pasadena, California, USA

## Abstract

Here, we present a draft genome sequence of Nak82, the second genome sequence available for the *Chloroflexi* class *Ardenticatenia* and the first from a sulfidic terrestrial hot spring. Nak82 is genetically and metabolically distinct from Ardenticatena maritima and likely represents a new genus- or family-level lineage lacking high-potential respiratory pathways.

## GENOME ANNOUNCEMENT

Ardenticatenia is a curious class in the *Chloroflexi* phylum; it is currently known only as a single isolate from an iron-rich hydrothermal field in Japan (1). Ardenticatena maritima is unique among the *Chloroflexi* for its capacity for iron reduction and complete denitrification (2). Here, we report the first genome sequence available from a second *Ardenticatenia* lineage strain, Nak82, recovered from Nakabusa Onsen in Japan. Nak82 is most closely related to Ardenticatena maritima but is genetically distinct at the genus or family level and does not share the diverse respiratory pathways that distinguish Ardenticatena maritima from other *Chloroflexi* species.

The Nak82 metagenome-assembled genome (MAG) was recovered from sequencing of Nakabusa Onsen, a moderately sulfidic hot spring in Japan. The site and metagenomic sequencing were described previously (3, 4). In brief, the site is a moderately sulfidic and alkaline (pH 8.5 to 9) hot spring with source water near 70°C and containing ∼0.1 mM sulfide (5). Samples were collected from microbial mats, and DNA was extracted and submitted to SeqMatic LLC (Fremont, CA) for sequencing with an Illumina HiSeq instrument. Sequences from four samples were coassembled with MEGAHIT v. 1.1.2 (6), and genome bins were constructed based on differential coverage using MetaBAT (7). Genome bins were assessed for completeness and contamination using CheckM (8) and uploaded to the RAST server for overall characterization (9).

The Nak82 MAG totals 3.49 Mb and consists of 2,942 protein-coding sequences across 195 contigs. The genome has a 58.7% GC content and is estimated by CheckM to be 91.74% complete, with 0.64% contamination. Forty-four tRNAs were recovered.

Phylogenetic analysis of Nak82 and other Chloroflexi using the RpoB protein—a valuable single-copy marker (10)—robustly places this organism as a sister taxon to Ardenticatena maritima; however, the RpoB sequences of these strains are only 72% similar, suggesting divergence to at least the genus level.

Nak82 does not have genes that encode the pathways for aerobic respiration and denitrification found in Ardenticatena maritima. The only dioxygen reductase recovered in the Nak82 genome is a *bd* oxidase, which may be used for oxygen detoxification, as it appears in obligate anaerobes, including some members of the phylum *Chloroflexi* class *Anaerolineae* (4, 11–14). This distribution of respiration genes is consistent with the acquisition of aerobic respiration and denitrification by Ardenticatena maritima via horizontal gene transfer after its divergence with Nak82, a pattern consistent with broader trends in the evolution of metabolic traits in the *Chloroflexi* (4, 15).

Genes involved in the synthesis of lipopolysaccharides and outer membrane proteins (e.g., *lpxB*, *omp85*, and *bamA*) were not recovered from Nak82. This is consistent with other evidence that members of the *Chloroflexi* lack an outer membrane, in contrast to members of their sister phylum *Armatimonadetes* (4, 16, 17).

### Accession number(s).

This whole-genome shotgun project was deposited in DDBJ/EMBL/GenBank under the accession number QEXY00000000.
